# Supplementary material for: The Isolation, Structural Characterization, and Biosynthetic Pathway of Unguisin from the Marine-Derived Fungus Aspergillus candidus
Source: Mar Drugs. 2025 May 21;23(5):219. doi: 10.3390/md23050219 (PMC12113358; doi:10.3390/md23050219)
Supplement: Supplementary file 1 [file marinedrugs-23-00219-s001.zip › marinedrugs-3628247-supplementary.pdf]

Supplementary Information

The Isolation, Structural Characterization, and Biosynthetic Pathway of Unguisin from Marine-Derived Fungus *Aspergillus candidus*

Wenjiao Diao[1,2,3,4†], Wei Zhang[1,3,4†], Xiaoxi Zhang[1,3,4], Siyu Du[1,3,4,5], Caijuan Zheng[2], Xuenian Huang[1,3,4,6]*, Xuefeng Lu[1,3,4,6,7,8]*

1 Shandong Provincial Key Laboratory of Synthetic Biology, Qingdao Institute of Bioenergy and Bioprocess Technology, Chinese Academy of Sciences, Qingdao 266101, China; diaowj@qibebt.ac.cn (W.D.), zhang_wei3@qibebt.ac.cn (W.Z.); zhangxiaoxi@qibebt.ac.cn (X.Z.); Dusy@qibebt.ac.cn (S.D.)

2 Key Laboratory of Tropical Medicinal Resource Chemistry of Ministry of Education, College of Chemistry and Chemical Engineering, Hainan Normal University, Haikou 571158, China; caijuan2002@163.com (C.Z.)

3 Shandong Energy Institute, Qingdao 266101, China;

4 Qingdao New Energy Shandong Laboratory, Qingdao 266101, China;

5 School of Chemistry and Chemical Engineering, University of Jinan, Jinan 250022, China;

6 University of Chinese Academy of Sciences, Beijing 100049, China;

7 Key Laboratory of Biofuels, Qingdao Institute of Bioenergy and Bioprocess Technology, Chinese Academy of Sciences, Qingdao, 266101, China;

8 Marine Biology and Biotechnology Laboratory, Qingdao National Laboratory for Marine Science and Technology, Qingdao 266237, China;

† W.D. and W.Z. contributed equally to this work

* Correspondence: huangxn@qibebt.ac.cn; lvxf@qibebt.ac.cn

Catalogue

Table S1. Cytotoxic activity evaluation of unguisins....................3
Table S2. Organization of the unguisins biosynthetic gene cluster *ugs* and the proposed functions of the genes....................4
Table S3. The homologous genes of *ungC* in *A.candidus* MEFC1001....................5
Table S4. Strains used in this study....................6
Table S5. ^{1}H and ^{13}C NMR data of compound 1 in DMSO-d_6 (600 and 150 MHz)....................7
Table S6. The primer sequences of gene knock-out used....................9
Figure S1. LC-MS/MS fragmentation spectrum of unguisin K (1) under the ESI negative ion mode.....................11
Figure S2. LC-MS analysis of L/D-FDAA derivatives of the hydrolysates of 1 and amino acid standards....................12
Figure S3. Gene knockout validation and the metabolite analysis of the *ugs* gene deletion mutants....................13
Figure S4. Enzymatic assays of UgsB using different substrates....................14
Figure S5. Phylogenetic analysis of UgsC....................15
Figure S6. The UV spectrum of unguisin K(1)....................16
Figure S7. The HRESIMS spectrum of 1....................17
Figure S8. The IR spectrum of 1....................18
Figure S9. The ^{1}H NMR spectrum of 1....................19
Figure S10. The DEPTQ^{13}C NMR spectrum of 1....................20
Figure S11. The ^{1}H-^{1}H COSY spectrum of 1....................21
Figure S12. The HSQC spectrum of 1....................22
Figure S13. The HMBC spectrum of 1....................23
Figure S14. The HRESIMS spectrum of 2....................24
Figure S15. The ^{1}H NMR spectrum of 2....................25
Figure S16. The DEPTQ^{13}C NMR spectrum of 2....................26
Figure S17. The HRESIMS spectrum of 3....................27
Figure S18. The ^{1}H NMR spectrum of 3....................28
Figure S19. The DEPTQ^{13}C NMR spectrum of 3....................29
Figure S20. The HRESIMS spectrum of 4....................30
Figure S21. The ^{1}H NMR spectrum of 4....................31
Figure S22. The DEPTQ^{13}C NMR spectrum of 4....................32

Table S1. Cytotoxic activity evaluation of unguisins

Cells \ Compounds	**1**	**2**	**3**	**4**	**Cisplatin**
**A549**	**5.19%±0.92%**	**2.53%±1.44%**	**2.79%±1.94%**	**5.15%±1.34%**	**90.21%±1.21%**
**MKN-45**	**8.12%±1.74%**	**6.17%±2.26%**	**3.68%±3.03%**	**7.12%±1.48%**	**90.21%±1.21%**
**Hela**	**5.31%±2.60%**	**3.03%±3.12%**	**4.24%±1.12%**	**2.68%±1.67%**	**98.99%±0.38%**
**K-562**	**4.61%±2.78%**	**4.61%±2.78%**	**3.18%±2.74%**	**3.32%±0.80%**	**67.49%±0.56%**
**MCF7**	**3.42%±3.84%**	**2.67%±2.96%**	**2.98%±2.05%**	**3.17%±1.27%**	**58.89%±0.84%**
**HepG2**	**58.89%±0.84%**	**2.22%±2.59%**	**4.90%±3.76%**	**4.53%±5.38%**	**92.55%±0.91%**
**293T**	**6.14%±3.06%**	**2.74%±3.80%**	**4.75%±2.50%**	**6.14%±2.61%**	**87.15%±2.53%**
**L-02**	**5.66%±2.77%**	**6.52%±2.26%**	**0.60%±3.75%**	**3.90%±3.72%**	**98.84%±0.49%**

The cytotoxic activity of unguisins **1-4** were evaluated against the normal human embryonic kidney and liver cells, as well as six cancer cells, using the CCK-8 assays at 50 μM. Data presented as mean±standard deviation of three replicate measurements of the same sample. Cisplatin was used as a positive control. The cell lines tested were human non-small cell lung cancer cells (A549), human gastric cancer cells (MKN-45), human cervical cancer cells (Hela), human chronic myelogenous leukemia cells (K-562), human breast cancer cells (MCF7), human hepatocellular carcinoma cells (HepG2), as well as human normal liver cells (L-02) and human embryonic kidney cells (293T).

Table S2. Organization of the unguisins biosynthetic gene cluster *ugs* and the proposed functions of the genes

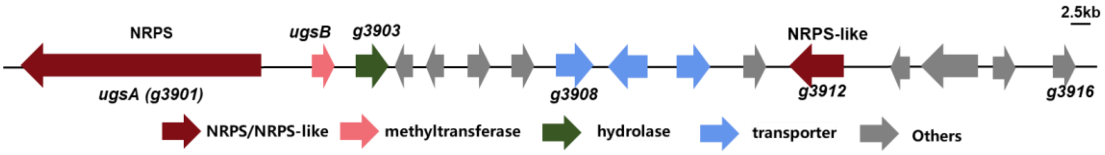

Gene ID	Homologous protein	Similarity/identity	Putative function
ugsA	*ungA'* *Aspergillus campestris* IBT 28561	91%/86%	NRPS
ugsB	*ungE'* *Aspergillus campestris* IBT 28561	97%/97%	Methyltransferase
g3903	*ungD'* *Aspergillus campestris* IBT 28561	89%/81%	Hydrolase
g3904	XP_024667707.1 *Aspergillus candidus*	97%/95%	Putative necrosis-inducing factor-domain-containing protein
g3905	XP_024694740.1 *Aspergillus campestris* IBT 28561	95%/91%	Uncharacterized protein
g3906	XP_024694743.1 *Aspergillus campestris* IBT 28561	87%/74%	Uncharacterized protein
g3907	XP_024694767.1 *Aspergillus campestris IBT 28561*	46%/39%	P-loop containing nucleoside triphosphate hydrolase protein
g3908	PLN79768.1 *Aspergillus taichungensis*	98%/97%	General substrate transporter
g3909	PLN79769.1 *Aspergillus taichungensis*	97%/96%	Major facilitator superfamily transporter
g3910	PLN79770.1 *Aspergillus taichungensis*	97%/96%	Synaptic vesicle transporter
g3911	XP_024694763.1 *Aspergillus campestris IBT 28561*	*97%/94%*	Uncharacterized protein
g3912	PLN79772.1 *Aspergillus taichungensis*	95%/93%	NRPS
g3913	PLN79774.1 *Aspergillus taichungensis*	100%/98%	Terrelysin
g3914	XP_024694759.1 *Aspergillus campestris IBT 28561*	98%/97%	Succinyl-CoA synthetase beta chain
g3915	XP_024694758.1 *Aspergillus campestris IBT 28561*	95%/93%	Enoyl-CoA hydratase/isomerase family protein
g3916	PLN79777.1 *Aspergillus taichungensis*	99%/99%	Uncharacterized protein

The gene cluster of unguisin were predicted by antiSMASH, and the functions of genes in the cluster were analyzed by BLASTP.

Table S3. The homologous genes of *ungC* in *A.candidus* MEFC1001

Gene	Similarity
g_1561	**71%**
g_15206	**46%**
g_3106	**41%**
g_1118	**41%**
g_2333	**38%**
g_949	**28%**
g_504	**30%**

Table S4. Strains used in this study

Strain	Description	Reference
MEFC1001	WT	Our lab
ΔugsA	MEFC1001: *ΔugsA(hph)*	This study
ΔugsB	MEFC1001: *Δku80(hph) ΔugsB(neo)*	This study
ΔugsC	MEFC1001: *Δku80(hph) ΔugsC(nat)*	This study
Δg3903	MEFC1001: *Δku80(hph) Δ3903(neo)*	This study
Δg3904	MEFC1001: *Δku80(hph) Δ3904(neo)*	This study
Δg3905	MEFC1001: *Δku80(hph) Δ3905(neo)*	This study
Δg3906	MEFC1001: *Δku80(hph) Δ3906(neo)*	This study
Δg3907	MEFC1001: *Δku80(hph) Δ3907(neo)*	This study
Δg3908	MEFC1001: *Δku80(hph) Δ3908(neo)*	This study
Δg3909	MEFC1001: *Δku80(hph) Δ3909(neo)*	This study
Δg3910	MEFC1001: *Δku80(hph) Δ3910(neo)*	This study
Δg3911	MEFC1001: *Δku80(hph) Δ3911(neo)*	This study
Δg3912	MEFC1001: *Δku80(hph) Δ3912(neo)*	This study
Δg3913	MEFC1001: *Δku80(hph) Δ3913(neo)*	This study
Δg3914	MEFC1001: *Δku80(hph) Δ3914(neo)*	This study
Δg3915	MEFC1001: *Δku80(hph) Δ3915(neo)*	This study
Δg3916	MEFC1001: *Δku80(hph) Δ3916(neo)*	This study
E. coli BL21-UgsC	UgsC protein expression strain	This study
E. coli BL21-UgsB	UgsB protein expression strain	This study

Table S5. ^{1}H and ^{13}C NMR data of compound 1 in DMSO-d_6 (600 and 150 MHz)

Position		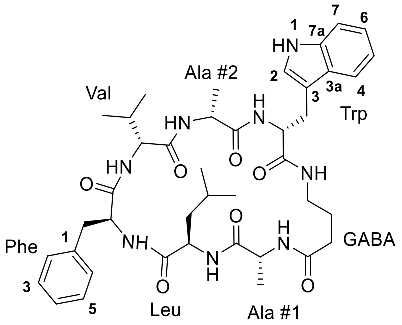	
		δ_H (mult, J in Hz)	δ_C (type)
Trp	C=O		171.0 (C)
	NH	8.20 (1H, d, 4.0)	
	α	3.76 (1H, m)	53.0 (CH)
	β	3.27 – 3.19 (2H, m)	24.9 (CH_2)
	NH-1	10.83 (1H, d, 2.6)	
	2	7.09 (1H, m)	123.7 (CH)
	3		110.7 (C)
	3a		127.1 (C)
	4	7.52 (1H, d, 7.9)	118.3 (CH)
	5	6.98 (1H, t, 7.4)	118.3 (CH)
	6	7.06 (1H, m)	121.0 (CH)
	7	7.33 (1H, d, 8.1)	111.4 (CH)
	7a		136.2 (C)
GABA	C=O		171.8 (C)
	NH	7.68 (1H, t, 5.4)	
	α	3.09 (1H, dq, 13.8, 7.0)<br>2.98 (tt, 12.9, 5.3)	38.7 (CH_2)
	β	1.71 (1H, dt, 14.1, 6.6)<br>1.57 (1H, dt, 13.7, 7.0)	25.8 (CH_2)
	γ	2.15 – 2.10 (1H, m)<br>1.95 (1H, dt, 13.4, 7.9)	33.0 (CH_2)
Ala #1	C=O		172.8 (C)
	NH	7.86 (1H, d, 5.5)	
	α	4.10 (1H, m)	48.1 (CH)
	β	1.14 (3H, q, 6.9)	17.7 (CH_3)
Ala #2	C=O		172.1 (C)
	NH	8.44 (1H, d, 4.6)	
	α	3.91 (1H, m)	49.9 (CH)
	β	1.14 (3H, q, 6.9)	17.1 (CH_3)

Continued above table

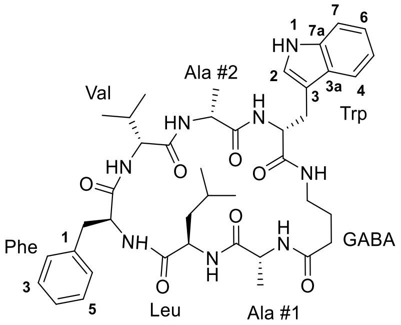

Position		δ_H (mult, *J* in Hz)	δ_C (type)
	C=O		172.6 (C)
	NH	7.98 (1H, d, 6.9)	
	α	4.00 (1H, m)	55.3 (CH)
Leu	β	1.23 (2H, pd, 14.3, 13.8, 6.1)	39.2 (CH_2)
	γ	0.83–0.77 (1H, m)	23.5 (CH)
	δ	0.60 (3H, d, 6.5)	22.9 (CH_3)
	δ′	0.71 (3H, d, 6.4)	22.1 (CH_3)
	C=O		
	NH	8.59 (1H, d, 8.4)	
	α	4.27(1H, m)	55.20(CH)
	β	3.27–3.19 (1H, m), 2.66–2.60 (1H, m)	36.30(CH_2)
	γ		
	1		138.65(C)
	2/6	7.25 – 7.18 (1H, m)	126.05(CH)
	3/5	7.25 – 7.18 (1H, m)	126.05(CH)
	4	7.15 (1H, tt, 5.5, 3.1)	128.02(CH)
	C=O		172.1(C)
	NH	7.78 (1H, d, 9.4)	
	α	4.06 (1H, m)	58.7(CH)
Val	β	2.06–1.98 (1H, m)	30.1(CH)
	γ	0.68 (3H, d, 6.6)	18.7(CH_3)
	γ′	0.78 (3H, d, 6.7)	18.8(CH_3)

Table S6. The primer sequences of gene knock-out used

Primers	Sequence (5'-3')	Notes
UugsA-F	atcagaccgttcatgtgaccc	Amplifying the upstream of ugsA
UugsA(hph)-R	ctttacgcttgcgatcccgaactcatccgtgaagtacagcac	
DugsA(hph)-F	cctgggttcgcaaagataattgatctctgctatggatcccacgc	Amplifying the downstream of ugsA
DugsA-R	gcaagggagcaggtctacaat	
ugsA-CF	cgcccaactgatcccttatct	Amplifying the knockout cassette of ugsA-hph
ugsA-CR	atgtctttgggctgctctagc	
UugsB-F	gcggcgtgaaagagaccatc	Amplifying the upstream of ugsB
UugsB(neo)-R	gaaaatctccagaggatcgccacgacggtgttctgctcc	
DugsB(neo)-F	Taaagaaggttacctctaaactggccccgggataactgc	Amplifying the downstream of ugsB
DugsB-R	gcgctgcgctgcatgtag	
ugsB-CF	ggtcccaaagccgctaagt	Amplifying the knockout cassette of ugsB-neo
ugsB-CR	ggaccggtgagacaggatgtc	
UugsC-F	cgagcgaatctttcagcagg	Amplifying the upstream of ugsC
UugsC(nat)-R	ttcaatatcagttaacgtcgcagcaggatcgaacaggagc	
DugsC(nat)-F	ttcaatatcagttaacgtcgcagcaggatcgaacaggagc	Amplifying the downstream of ugsC
DugsC-R	ggtagaccaccgggtacac	
ugsC-CF	gtcaaagtccggtctcgcg	Amplifying the knockout cassette of ugsC-nat
ugsC-CR	acggcttagctccaatgacag	
Ug3903-F	cgatagactcgtcttccg	Amplifying the upstream of 3903
Ug3903(neo)-R	gaaaatctccagaggatcgccaggatgtcctctatcgtg	
Dg3903(neo)-F	taaagaaggttacctctaaaccttgtgaagctcactgtcgc	Amplifying the downstream of 3903
Dg3903-R	ccaactgccaactgttatcg	
g3903-CF	gatgggacacgacttttggc	Amplifying the knockout cassette of 3903-neo
g3903-CR	gctttgaaaacgcgagcacc	
Ug3904-F	ctgtgactaggctgtttg	Amplifying the upstream of 3904
Ug3904(neo)-R	gaaaatctccagaggatcgcaatcgtccggcagtcgtc	
Dg3904(neo)-F	taaagaaggttacctctaaaccgataggtttgagtggaagg	Amplifying the downstream of 3904
Dg3904-R	gtgacagaagggacacttcg	
g3904-CF	gtccgaaaacgcagtactac	Amplifying the knockout cassette of 3904-neo
g3904-CR	ggagtatgtcaaaaccgctc	
Ug3905-F	cactggccccagaccaat	Amplifying the upstream of 3905
Ug3905(neo)-R	gaaaatctccagaggatcgcgatatgttggcaatcggtg	
Dg3905(neo)-F	taaagaaggttacctctaaaccgaaattcggcagacctg	Amplifying the downstream of 3905
Dg3905-R	gctatgtgttcaacggag	
g3905-CF	gccattgcactgcctccatttc	Amplifying the knockout cassette of 3905-neo
g3905-CR	gctggttgttcagaggcttgg	
Ug3906-F	cagtctcatgttccgggc	Amplifying the upstream of 3906
Ug3906(neo)-R	gaaaatctccagaggatcgccatcttggcgacgatatgg	
Dg3906(neo)-F	taaagaaggttacctctaaaccgcagccgcaatattctt	Amplifying the downstream of 3906
Dg3906-R	gtctggcgatgaaatagc	
g3906-CF	ggttgaaacttccaacgtgg	Amplifying the knockout cassette of 3906-neo
g3906-CR	gatcccgaccaagatttcc	
Ug3907-F	cacggatgtgaccgtctg	Amplifying the upstream of 3907
Ug3907(neo)-R	gaaaatctccagaggatcgcgaagtactcgagaatctggg	
Dg3907(neo)-F	taaagaaggttacctctaaaccgaccaagctgtctgccg	Amplifying the downstream of 3907
Dg3907-R	gctgagcatagccacatg	
g3907-CF	gttgattcaaacagcgctgc	Amplifying the knockout cassette of 3907-neo
g3907-CR	ggtaggaactgccgcgtc	
Ug3908-F	ggtttaggtctgatcaccgc	Amplifying the upstream of 3908
Ug3908(neo)-R	gaaaatctccagaggatcgcggtgatgatcgagcccac	
Dg3908(neo)-F	taaagaaggttacctctaaacgaggcccttgacacctac	Amplifying the downstream of 3908
Dg3908-R	gctcgctcctccatttcatc	
g3908-CF	gagcctcggccaaattgc	Amplifying the knockout cassette of 3908-neo
g3908-CR	cacgacgaccggaacgatc	
Ug3909-F	cgatagcagactagccgt	Amplifying the upstream of 3909
Ug3909(neo)-R	gaaaatctccagaggatcgcgatcgccaggtattgcag	

Primers	Sequence (5'-3')	Notes
Dg3909(neo)-F	taaagaaggttacctctaaaccgacgaaatctggagcgc	Amplifying the downstream of 3909
Dg3909-R	gctgggtgtccagtccac	
g3909-CF	cctgtctggtgcaatggaggc	Amplifying the knockout cassette of 3909-neo
g3909-CR	gcaggtcctgatgatgggag	
Ug3910-F	gatgaacgtgtcgattcacc	Amplifying the upstream of 3910
Ug3910(neo)-R	gaaaatctccagaggatcgcctcgacgacatactcctccc	
Dg3910(neo)-F	taaagaaggttacctctaaacccagcgacatcccgtggatt	Amplifying the downstream of 3910
Dg3910-R	cacacctggtccagccagg	
g3910-CF	gcaccagacaggcttctctat	Amplifying the knockout cassette of 3910-neo
g3910-CR	ctggatatcaaagcttcgctc	
Ug3911-F	gctgaacgctctatcgatac	Amplifying the upstream of 3911
Ug3911(neo)-R	gaaaatctccagaggatcgccgtcaccagtaggagtaagc	
Dg3911(neo)-F	taaagaaggttacctctaaacctgtattcctccatggcctg	Amplifying the downstream of 3911
Dg3911-R	cgacggtgaggccaactatt	
g3911-CF	ttgcgcgttgaggactcac	Amplifying the knockout cassette of 3911-neo
g3911-CR	gcaccatctgggtgggcag	
Ug3912-F	gttgcgcaggactcctaacg	Amplifying the upstream of 3912
Ug3912(neo)-R	gaaaatctccagaggatcgcgccgacggtctcctggagtg	
Dg3912(neo)-F	taaagaaggttacctctaaacgctcaccagcagcaccgt	Amplifying the downstream of 3912
Dg3912-R	gcatagtactggttgcggcg	
g3912-CF	gatagatgccctggacggtg	Amplifying the knockout cassette of 3912-neo
g3912-CR	cgaacggcgatttagtgggc	
Ug3913-F	cctggcggaggagtacgagg	Amplifying the upstream of 3913
Ug3913(neo)-R	gaaaatctccagaggatcgcgtcatcgggagtgtggaagg	
Dg3913(neo)-F	taaagaaggttacctctaaacgaatacagcggatggagcc	Amplifying the downstream of 3913
Dg3913-R	gcatagacaccacactcgg	
g3913-CF	gtttgatctggacgacgctg	Amplifying the knockout cassette of 3913-neo
g3913-CR	ctggcggtggaagcaattg	
Ug3914-F	gtcttgcgtgtgggaccac	Amplifying the upstream of 3914
Ug3914(neo)-R	gaaaatctccagaggatcgcgtgacggcgacgtaatac	
Dg3914(neo)-F	taaagaaggttacctctaaacgccgtggagatgctgaac	Amplifying the downstream of 3914
Dg3914-R	ggctccatccgctgtattc	
g3914-CF	gatctgcactagcttgtcagc	Amplifying the knockout cassette of 3914-neo
g3914-CR	gcagctcgcagatcttgg	
Ug3915-F	ccttcccgcactatgacatg	Amplifying the upstream of 3915
Ug3915(neo)-R	gaaaatctccagaggatcgcccatgtcgaggtcggctc	
Dg3915(neo)-F	taaagaaggttacctctaaacgtcggtccctcctttgggag	Amplifying the downstream of 3915
Dg3915-R	gtcggagaacgagcgtcag	
g3915-CF	ccatcgtcaatctgctggcc	Amplifying the knockout cassette of 3915-neo
g3915-CR	caccgtgcatgcgtaggac	
Ug3916-F	ggacgatccggtggggag	Amplifying the upstream of 3916
Ug3916(neo)-R	gaaaatctccagaggatcgcgctgacctcgacctgtcg	
Dg3916(neo)-F	taaagaaggttacctctaaacgctctcgccttcatggag	Amplifying the downstream of 3916
Dg3916-R	catgcccgctcggaacgc	
g3916-CF	cattggaagtagcagctccgg	Amplifying the knockout cassette of 3916-neo
g3916-CR	ccgacacaagggtggattcg	

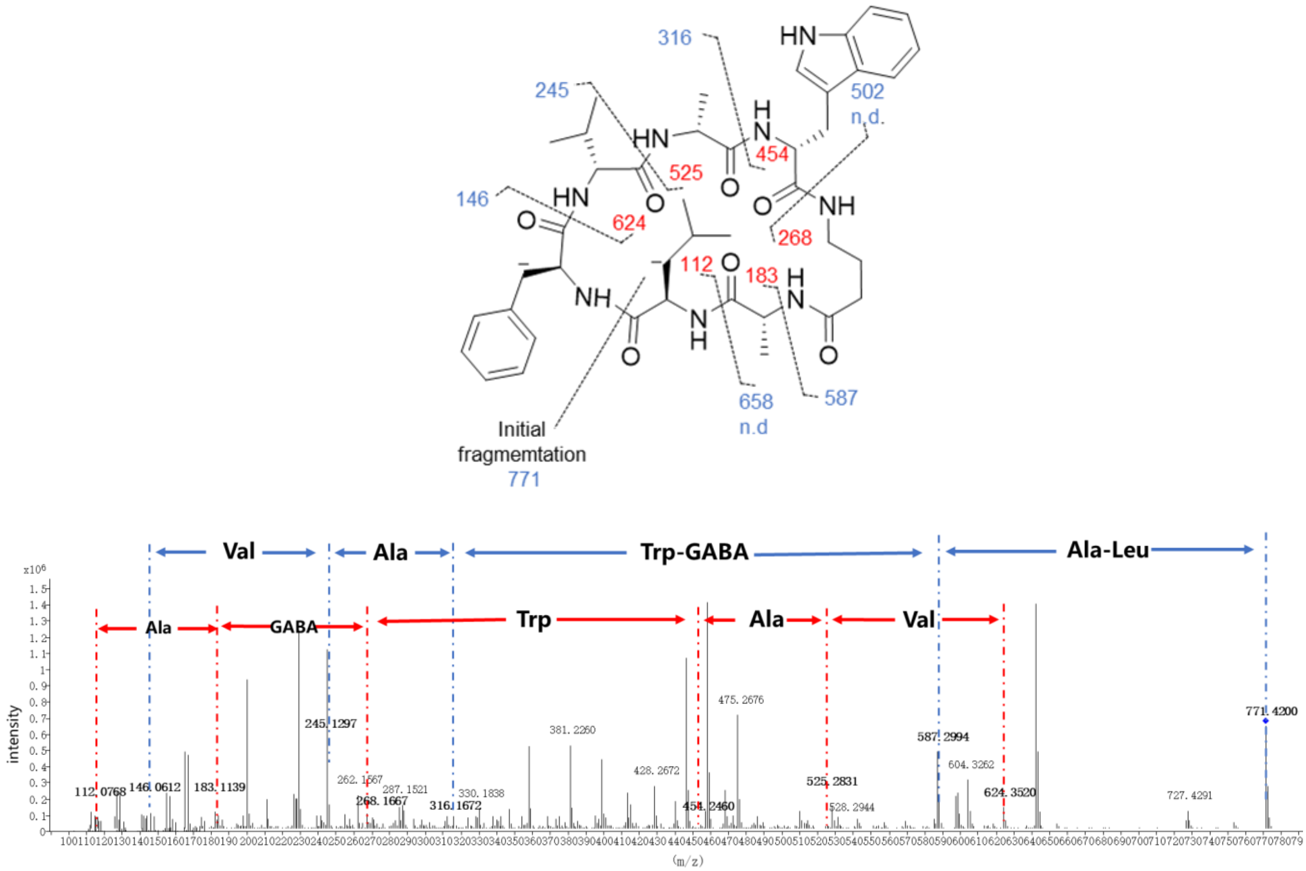

Precursor ion [M-H]⁻	Precursor ion mass (m/z) calcd.	Precursor ion mass (m/z) obsd.	Product ion mass (m/z)	formula of the product ion	amino acids
$C_{41}H_{55}N_8O_7^-$	771.4199	771.4200	112.0768	$C_6H_{10}NO^-$	Leu
			183.1139	$C_9H_{15}N_2O_2^-$	Leu+Ala
			245.1297	$C_{14}H_{17}N_2O_2^-$	Phe+Val
			268.1667	$C_{13}H_{22}N_3O_3^-$	Leu+Ala+GABA
			316.1672	$C_{17}H_{22}N_3O_3^-$	Phe+Val+Ala
			587.2994	$C_{32}H_{39}N_6O_5^-$	Phe+Val+Ala+Trp+GABA
			624.3520	$C_{32}H_{46}N_7O_6^-$	Leu+Ala+GABA+Trp+Ala +Val

Figure S1. LC-MS/MS fragmentation spectrum of unguisin K (1) under the ESI negative ion mode.

n.d.: Not detected.

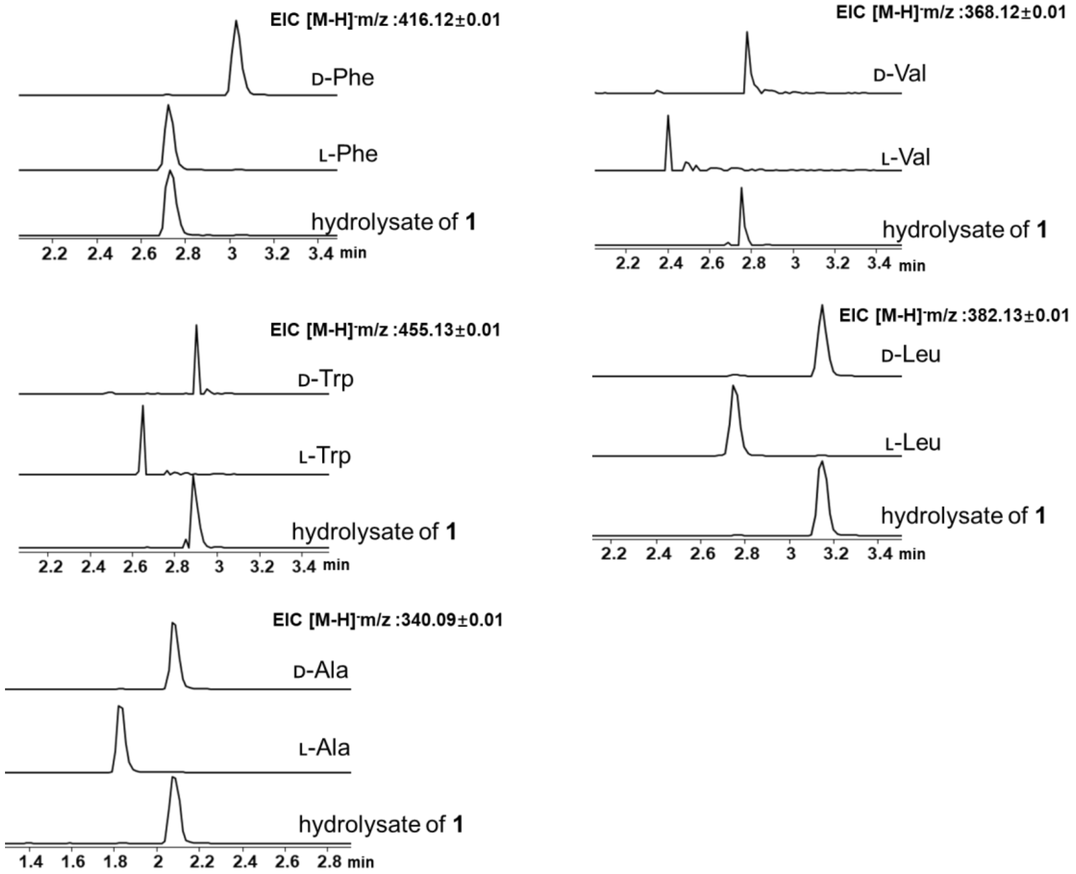

Figure S2. LC-MS analysis of L/D-FDAA derivatives of the hydrolysates of 1 and amino acid standards.

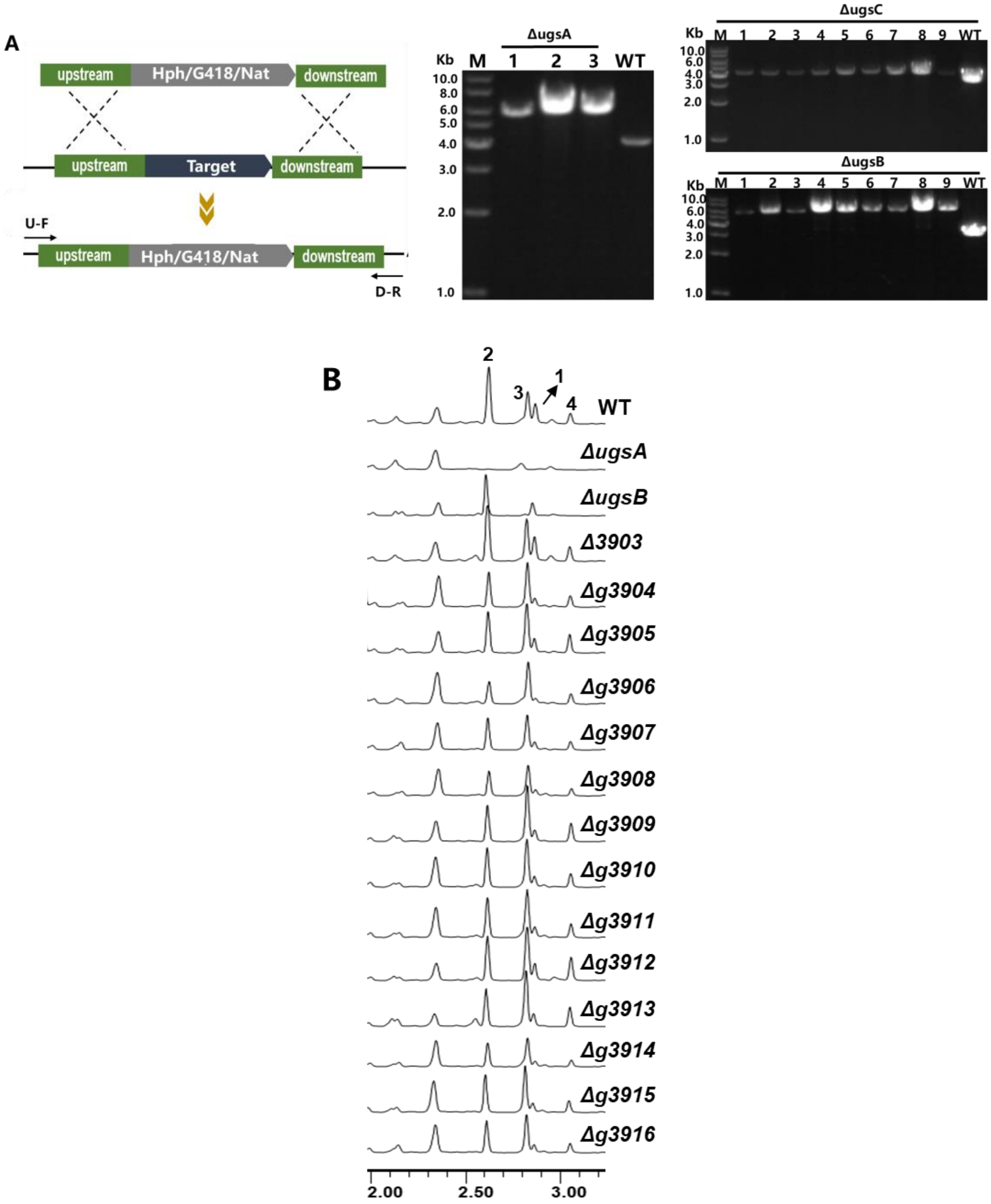

Figure S3. Gene knockout validation and the metabolite analysis of the *ugs* gene deletion mutants

A: Schematic diagram of gene knockout and the verification of the knockout gene of *ugsA*, *ugsB* and *ugsC*. (deletion *ugsA* using *hph* as a selection marker; deletion of *ugsB* using *G418* as a selection marker; deletion of *ugsC* using *Nat* as a selection marker)

B: HPLC profiles of extracts from the *ugs* gene deletion mutants.

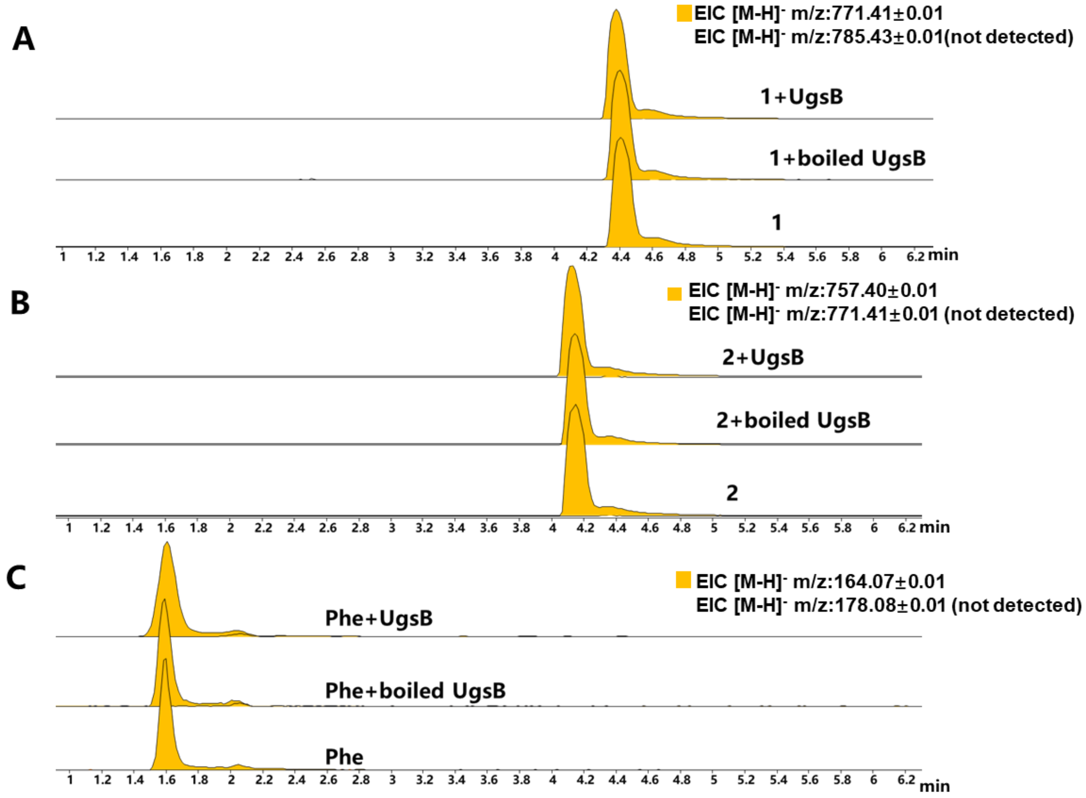

Figure S4. Enzymatic assays of UgsB using different substrates

A: Compound **1** as the substrate and its methylated product ([M-H]$^-$ m/z:785.43±0.01) was not detected. B: Compound **2** as the substrate and its methylated product ([M-H]$^-$ m/z:771.41±0.01) was not detected. C: Phe as the substrate and its methylated product ([M-H]$^-$ m/z:178.08±0.01) was not detected.

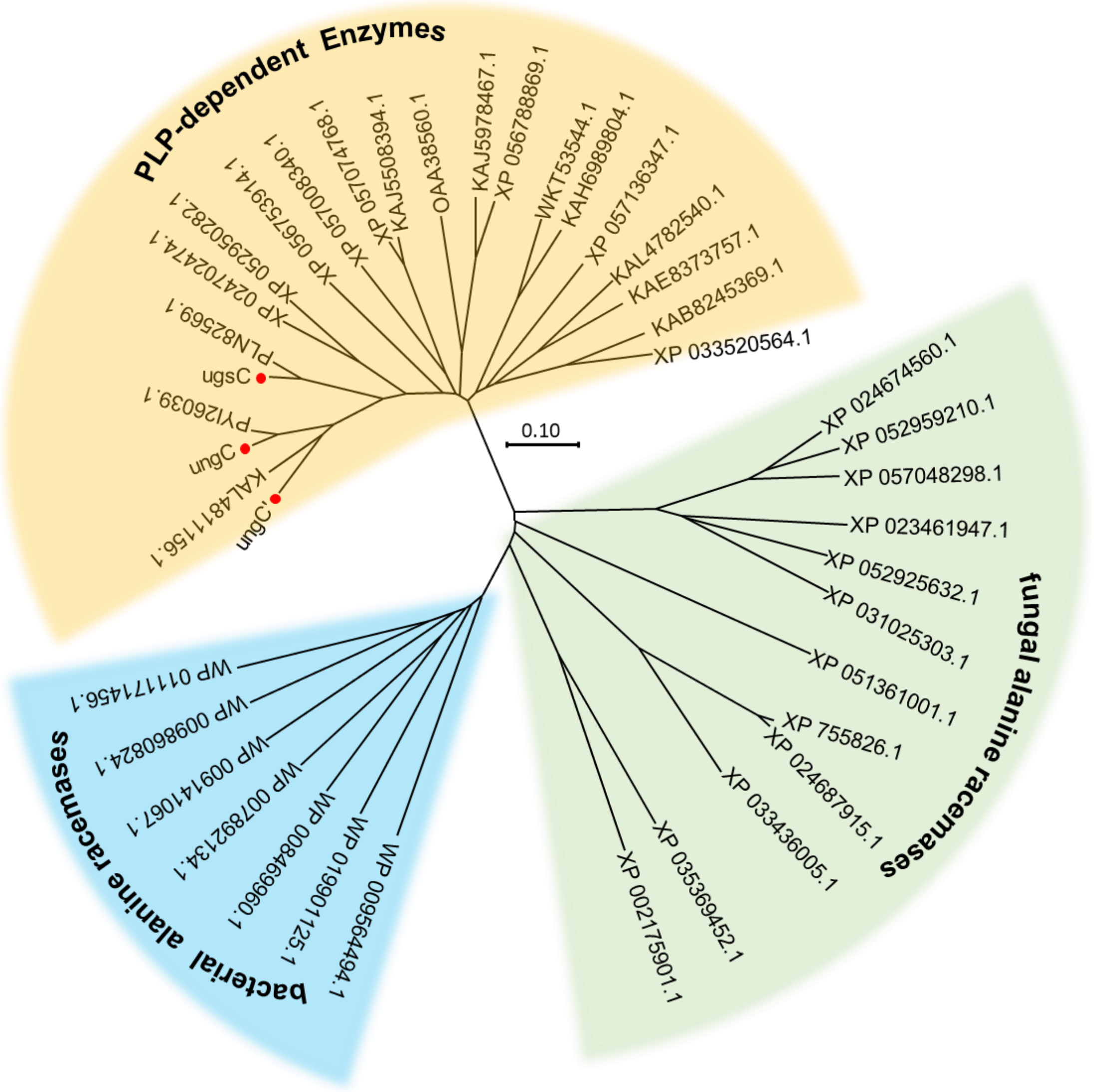

Figure S5. Phylogenetic analysis of UgsC

The amino acid sequences were downloaded from NCBI database. The phylogenetic tree was constructed using the neighbor-joining method based on ClustalW multiple alignment using MEGA 7.0.14 software. Bootstrap values calculated for 500 replications are shown. The red dots represent the Ala racemase enzymes that have been reported previously in the biosynthesis of unguisins.

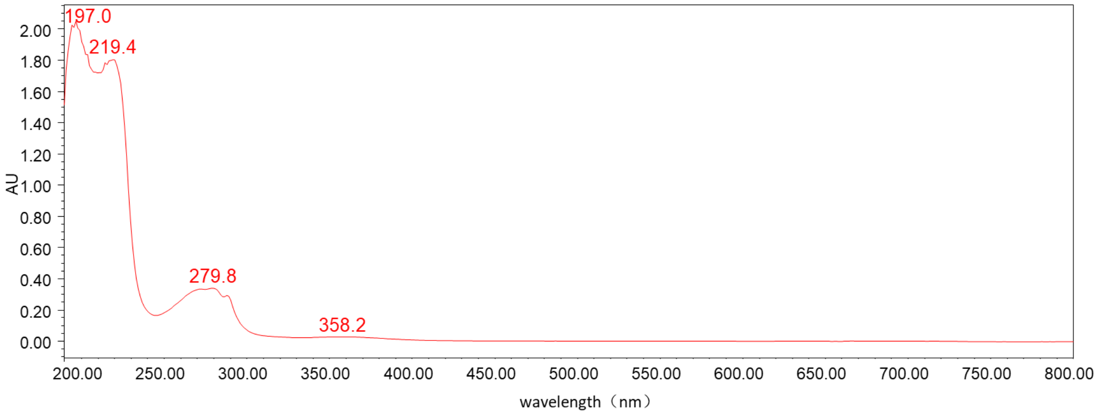

Figure S6. The UV spectrum of unguisin K (1)

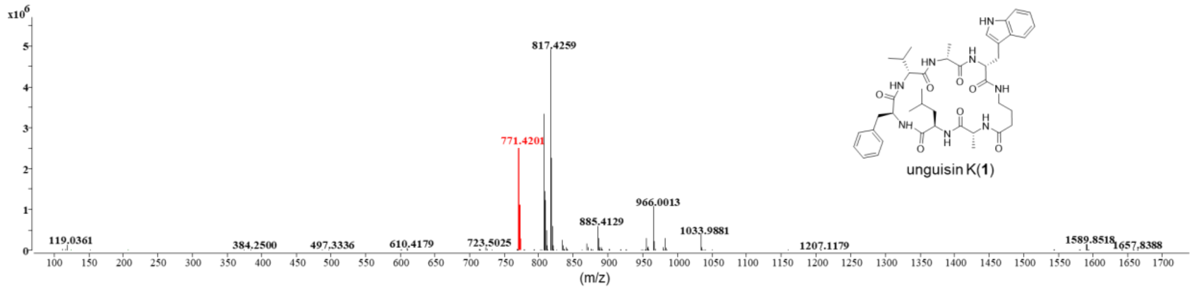

Figure S7. The HRESIMS spectrum of 1

The HRESIMS spectrum (negative) showed m/z 771.4201 $[M-H]^-$ (calcd. for $C_{41}H_{55}N_8O_7$, 771.4199).

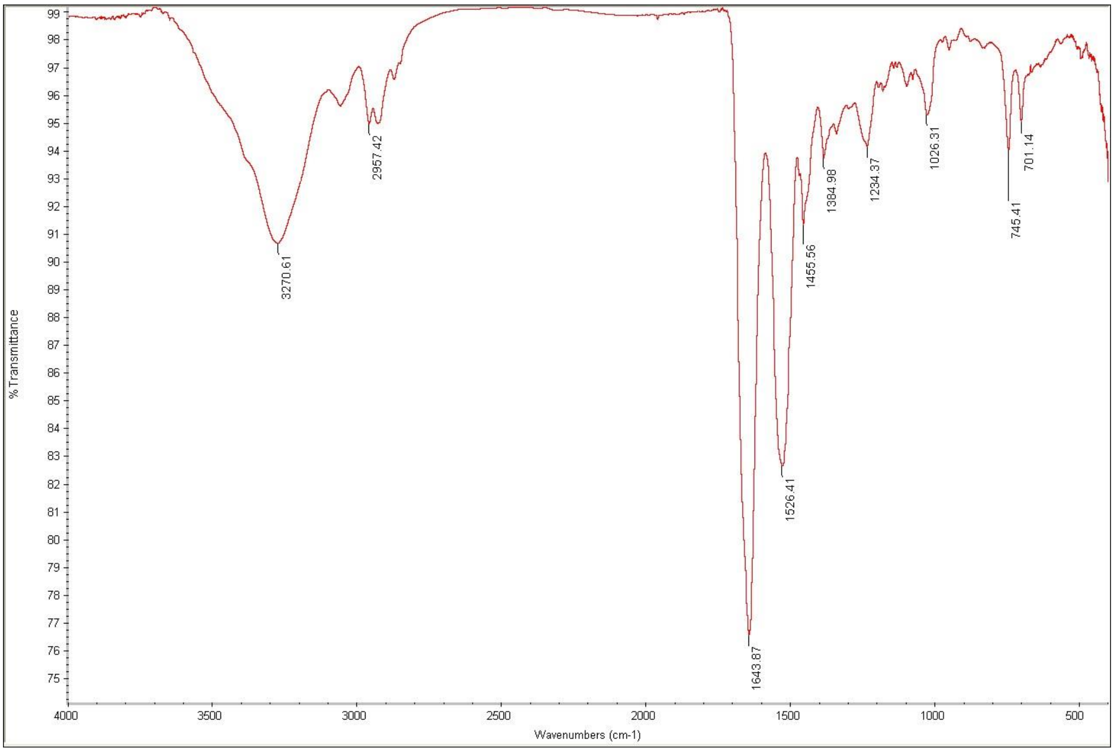

Figure S8. The IR spectrum of 1

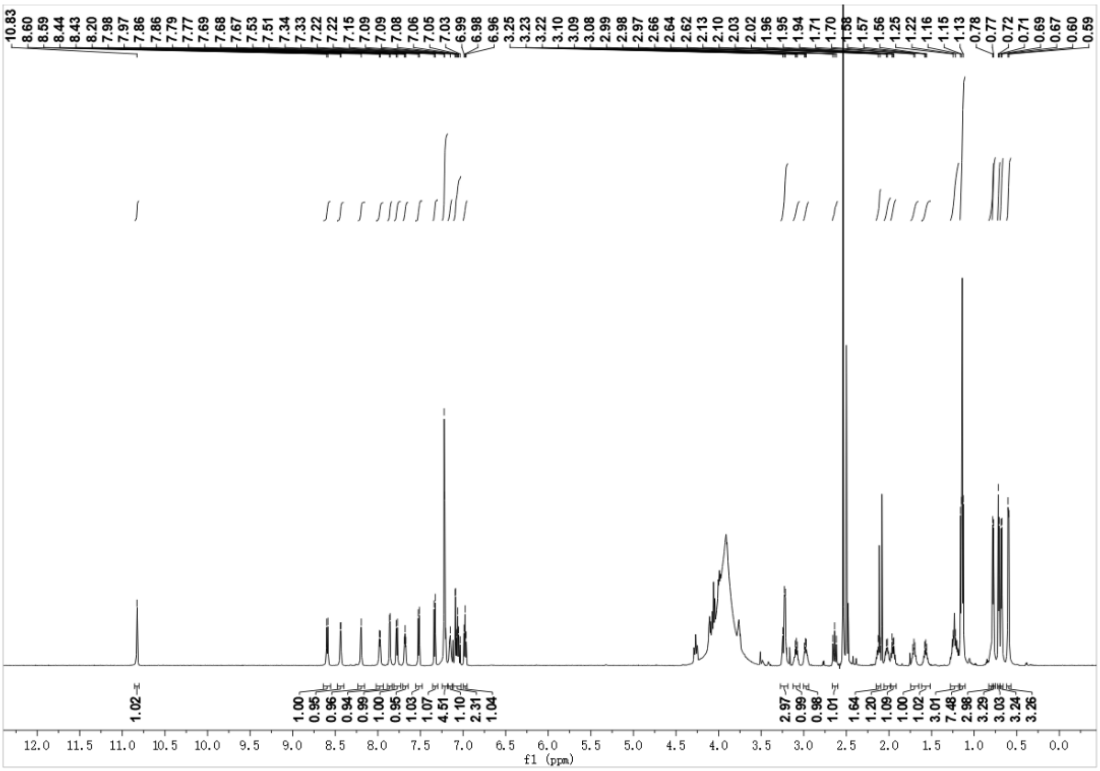

Figure S9. The ^{1}H NMR spectrum of 1

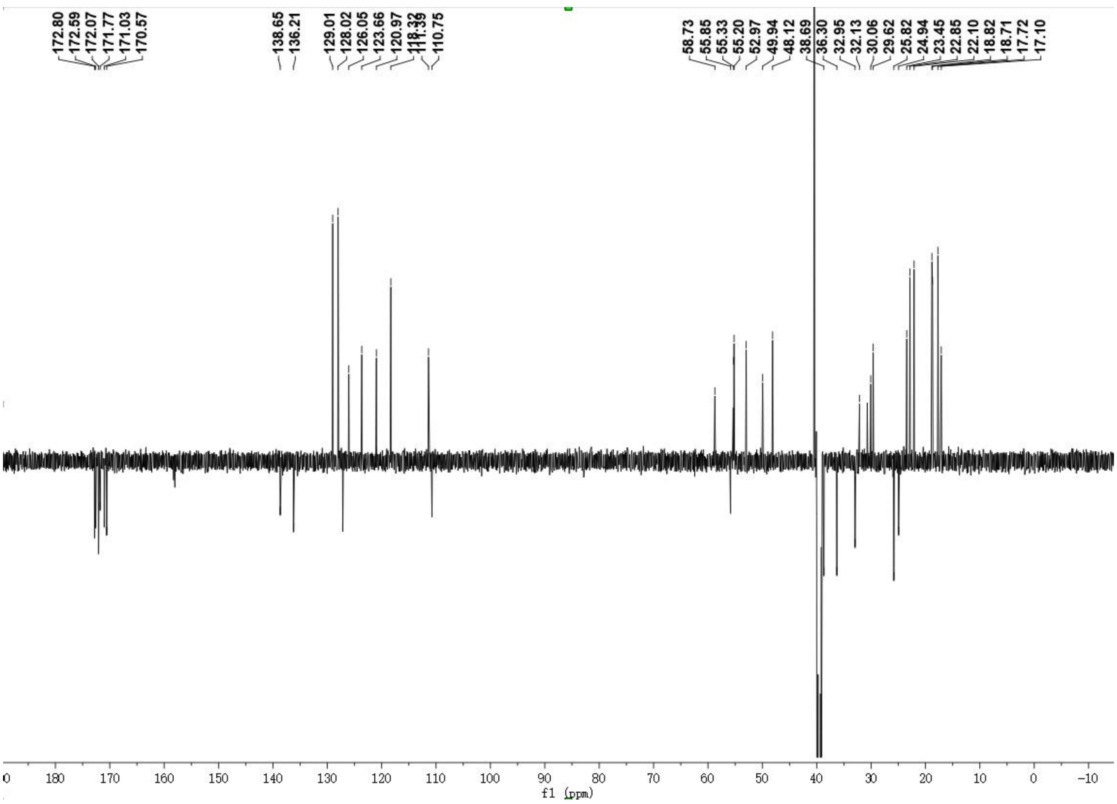

Figure S10. The DEPTQ^{13}C NMR spectrum of 1

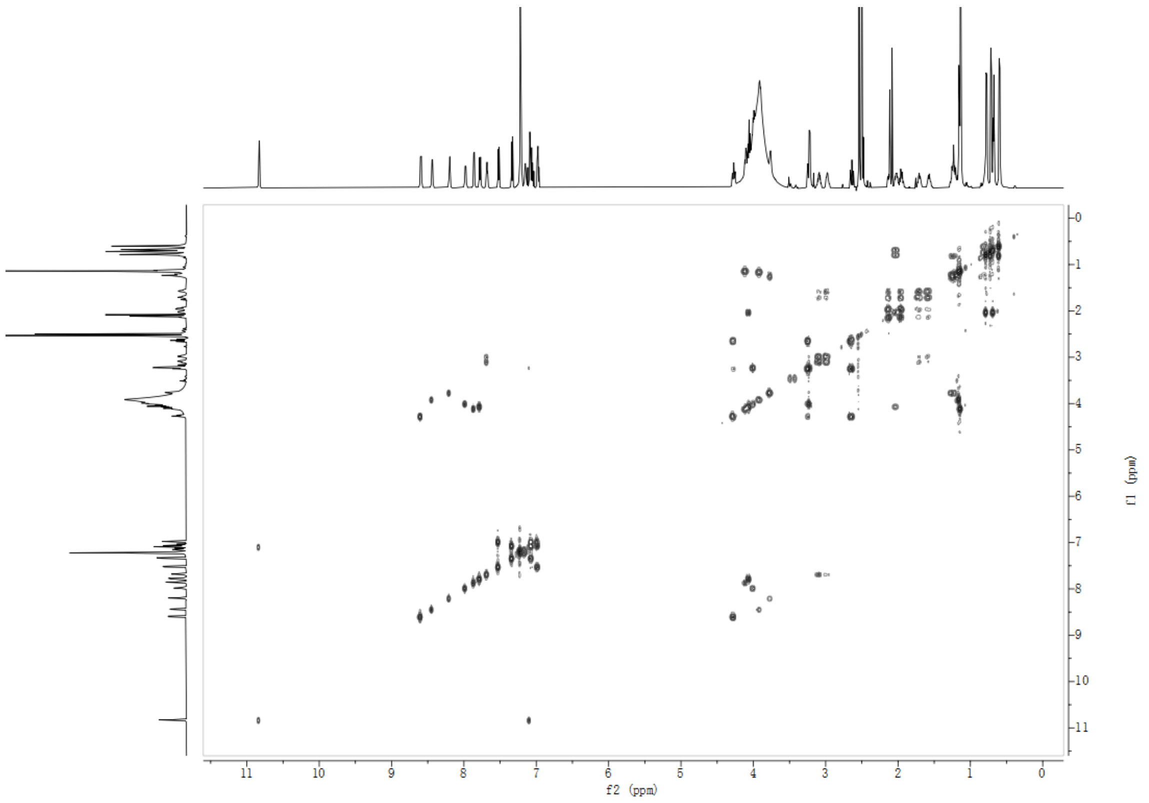

Figure S11. The ^1H-^1H COSY spectrum of 1

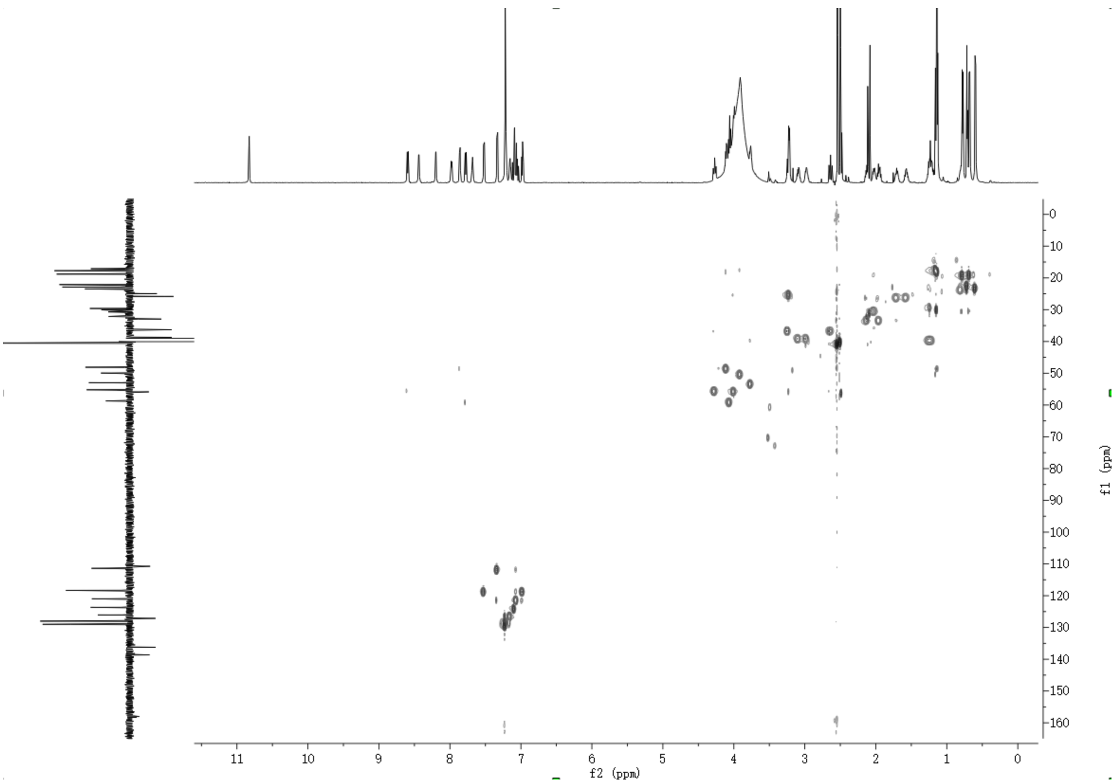

Figure S12. The HSQC spectrum of 1

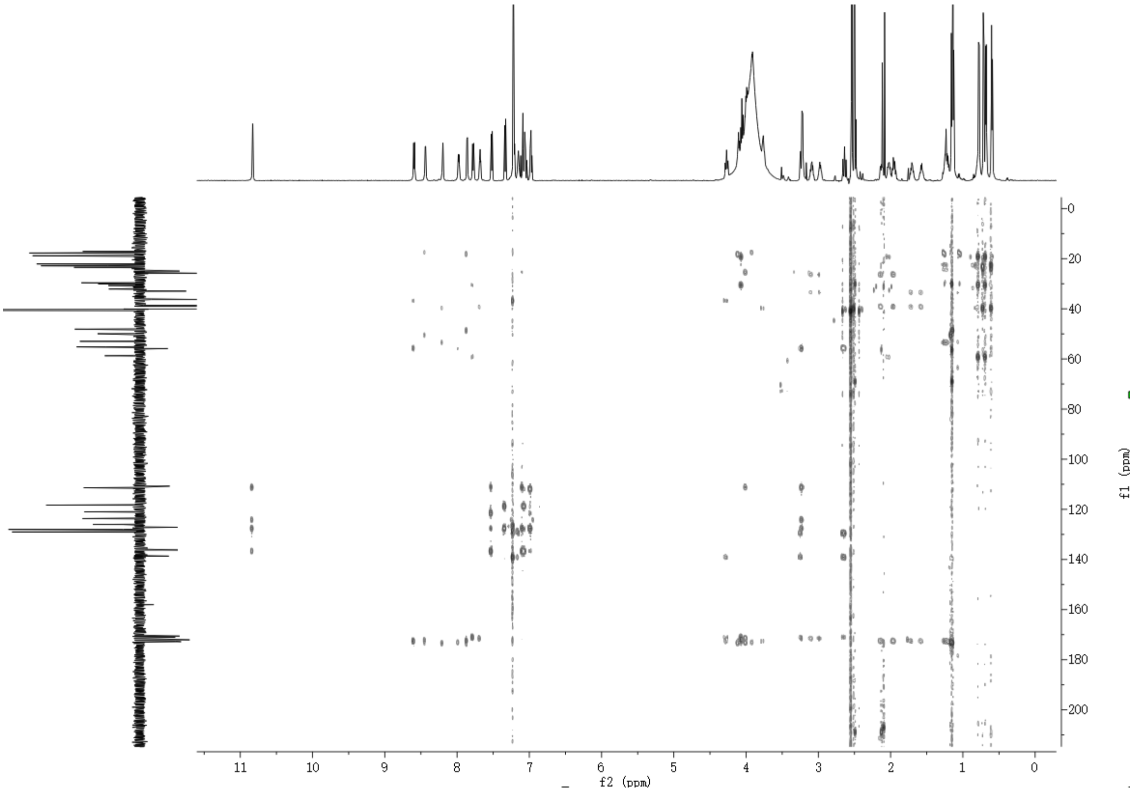

Figure S13. The HMBC spectrum of 1

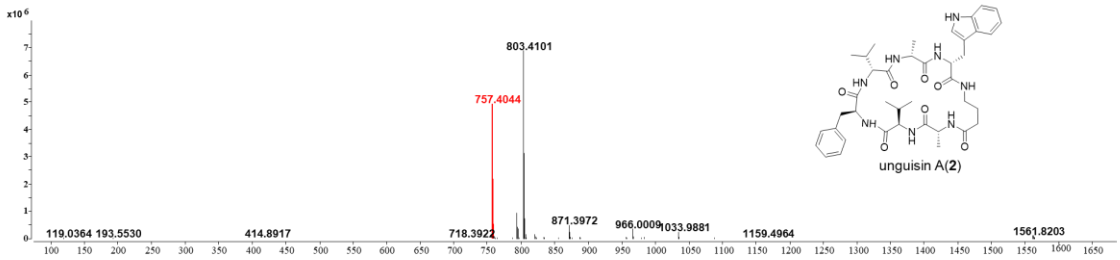

Figure S14. The HRESIMS spectrum of 2

The HRESIMS spectrum (negative) showed m/z 757.4044 [M-H]$^-$ (calcd. for $C_{40}H_{53}N_8O_7$, 757.4043).

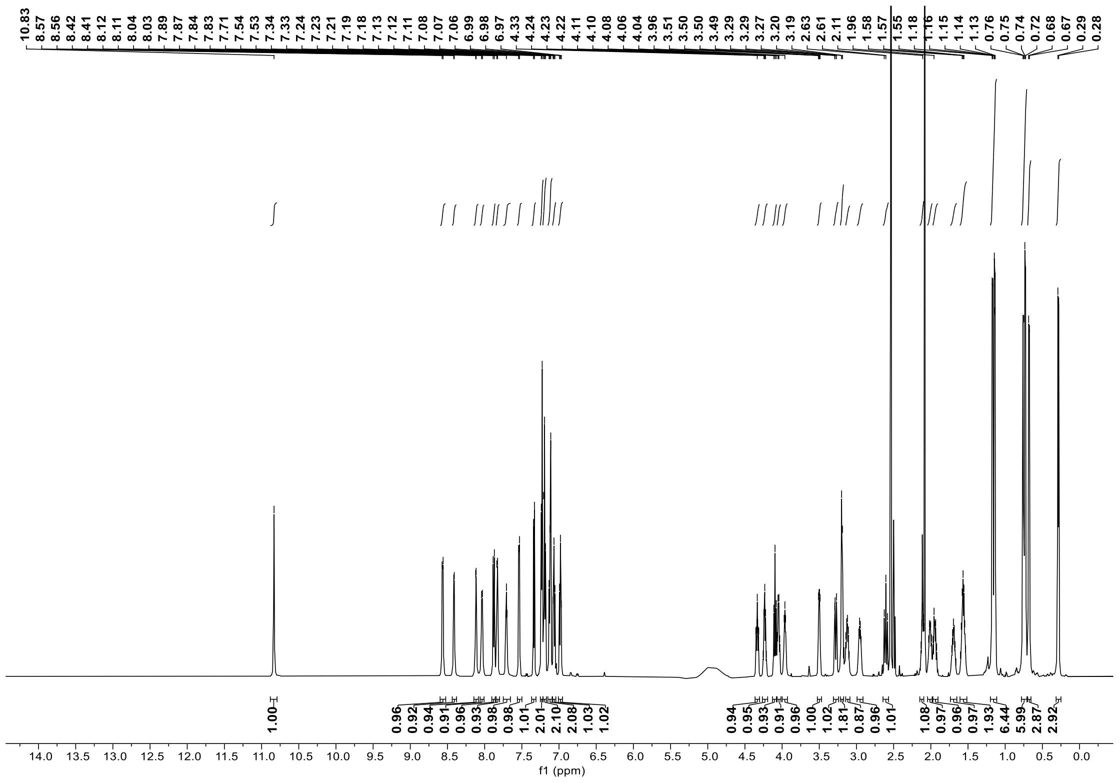

Figure S15. The ^1H NMR spectrum of 2

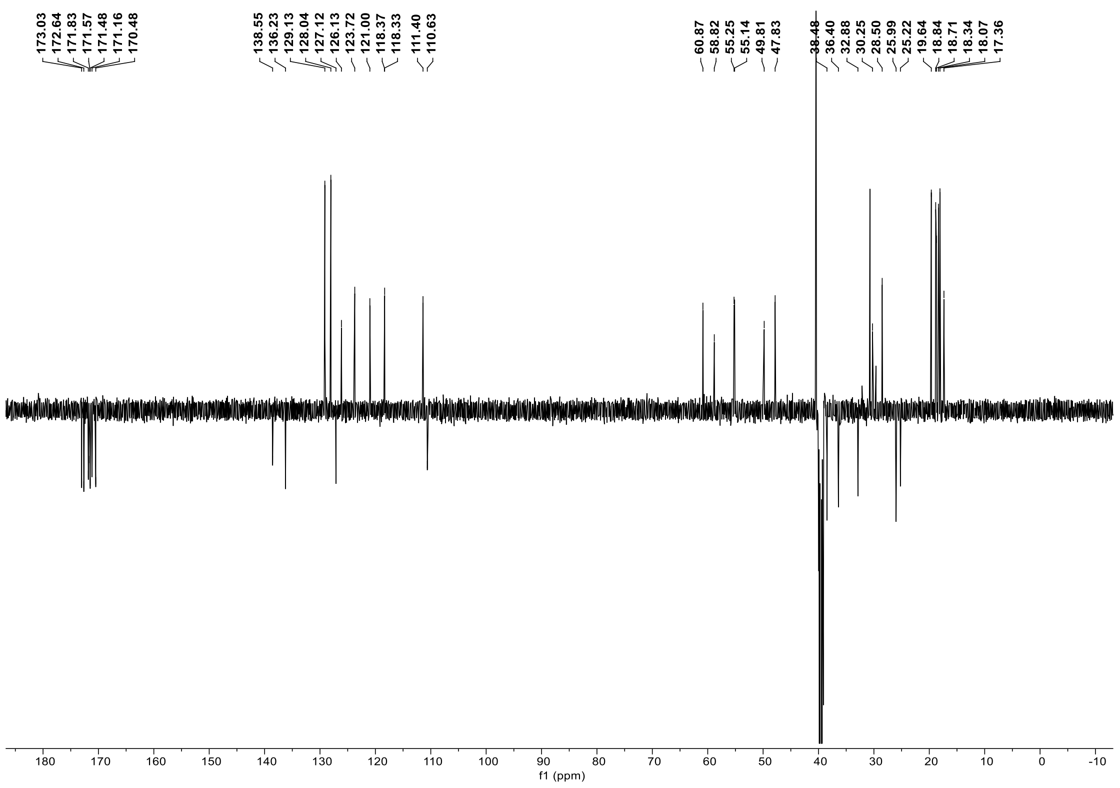

Figure S16. The DEPTQ^{13}C NMR spectrum of 2

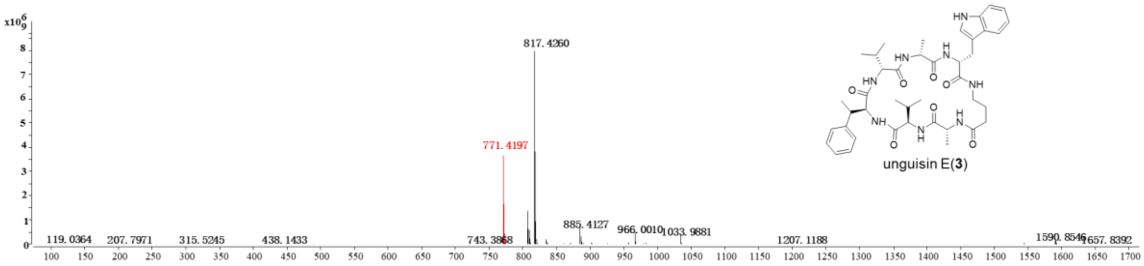

Figure S17. The HRESIMS spectrum of 3

The HRESIMS spectrum (negative) showed m/z 771.4197 $[M-H]^-$ (calcd. for $C_{41}H_{55}N_8O_7$, 771.4199).

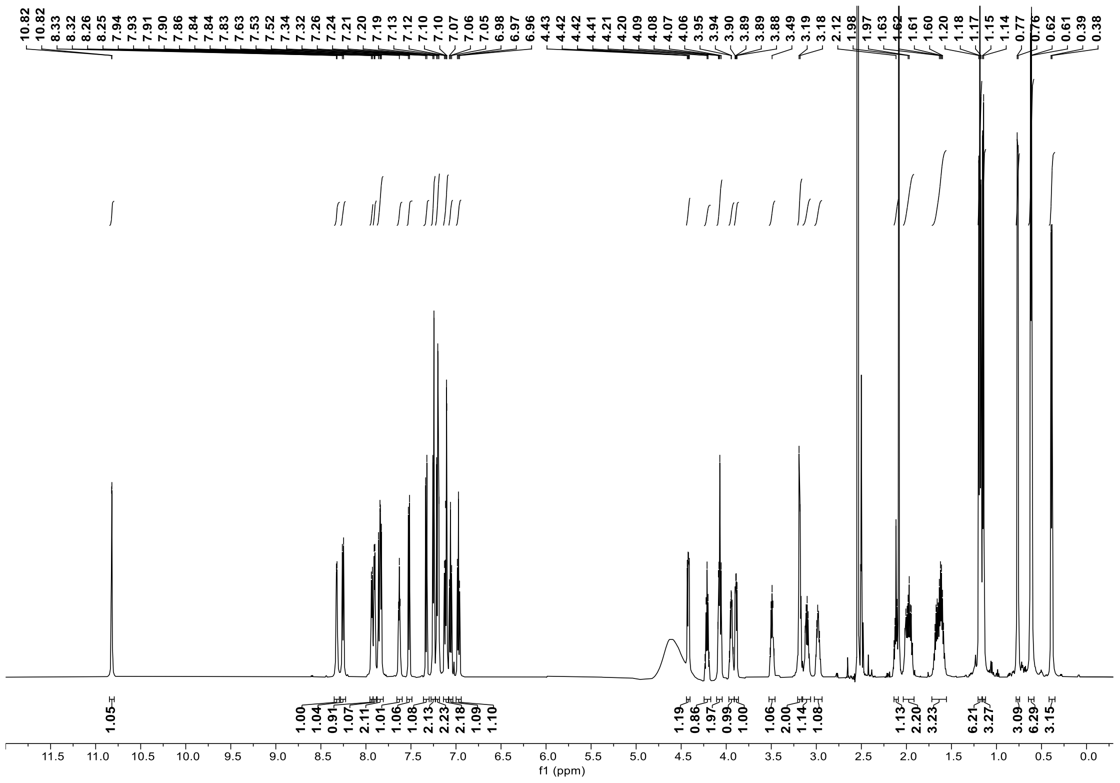

Figure S18. The ^{1}H NMR spectrum of 3

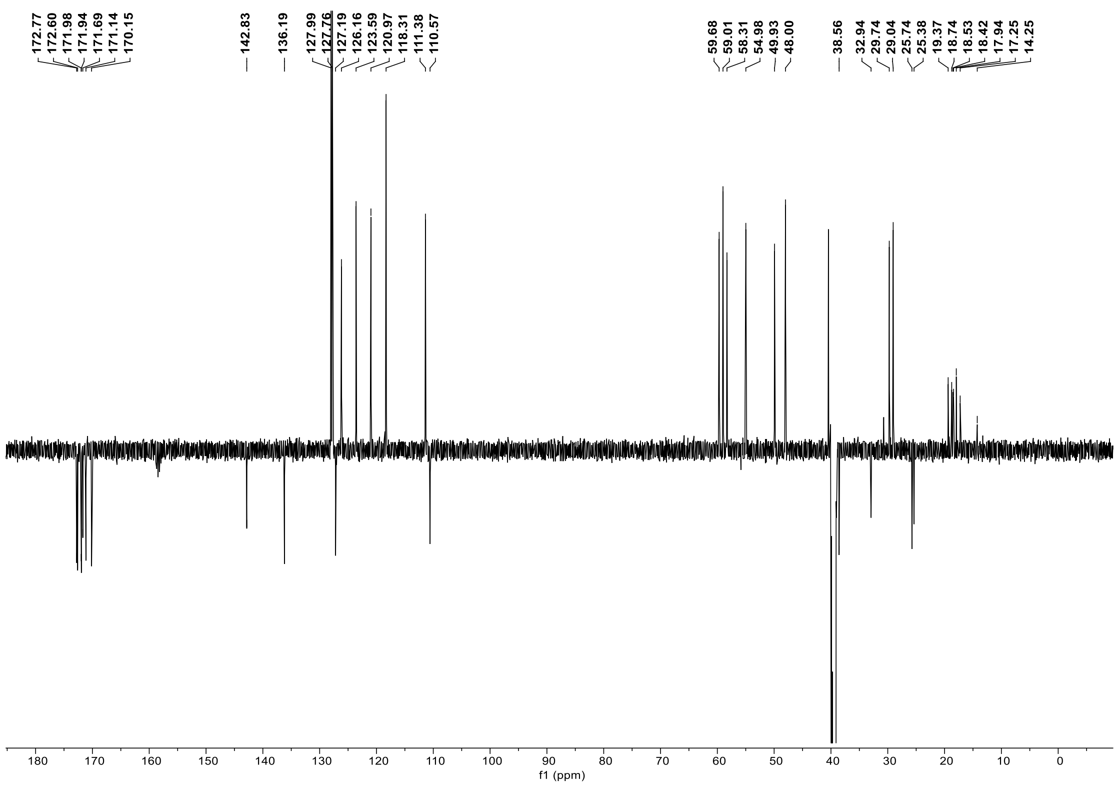

Figure S19. The DEPTQ13C NMR spectrum of 3

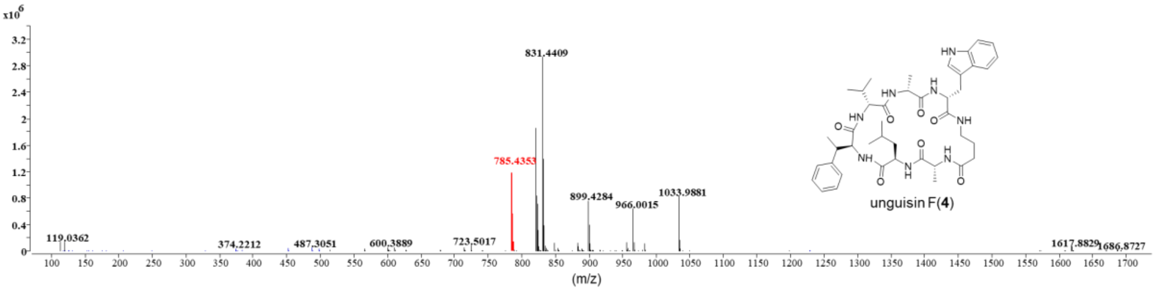

Figure S20. The HRESIMS spectrum of 4

The HRESIMS spectrum (negative) showed m/z 785.4353 $[M-H]^-$ (calcd. for $C_{42}H_{57}N_8O_7$, 785.4356).

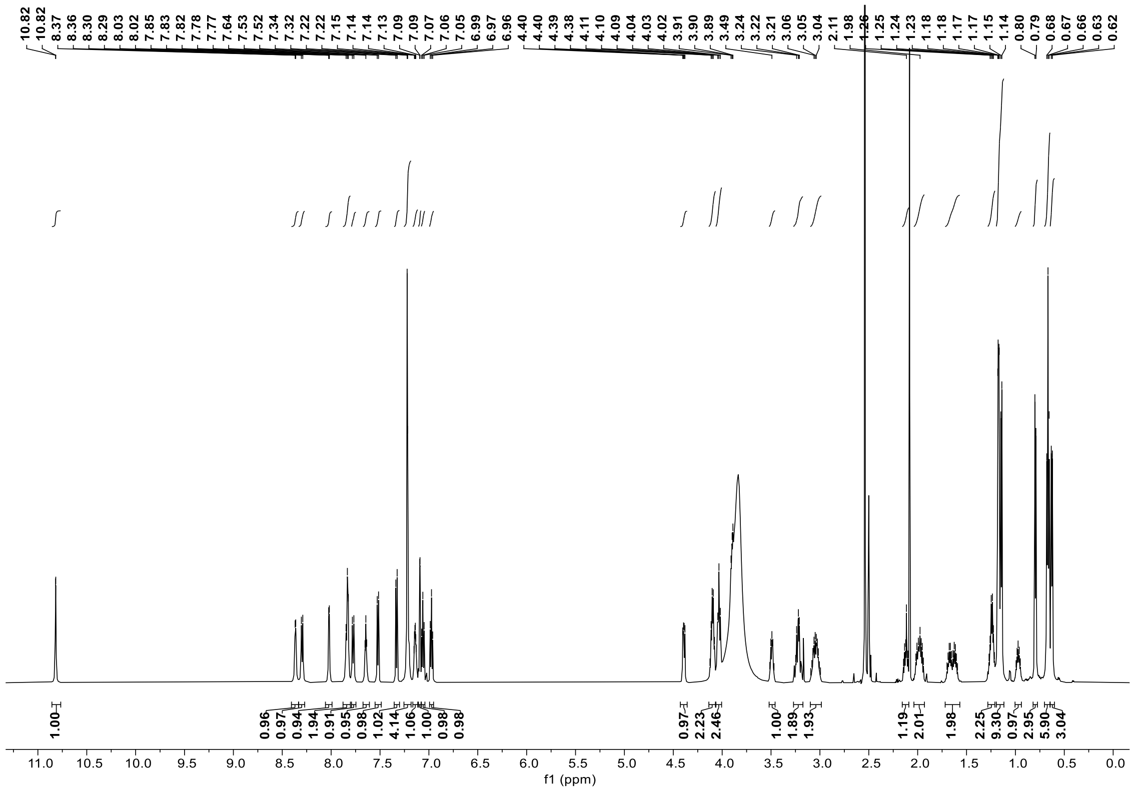

Figure S21. The ^{1}H NMR spectrum of 4

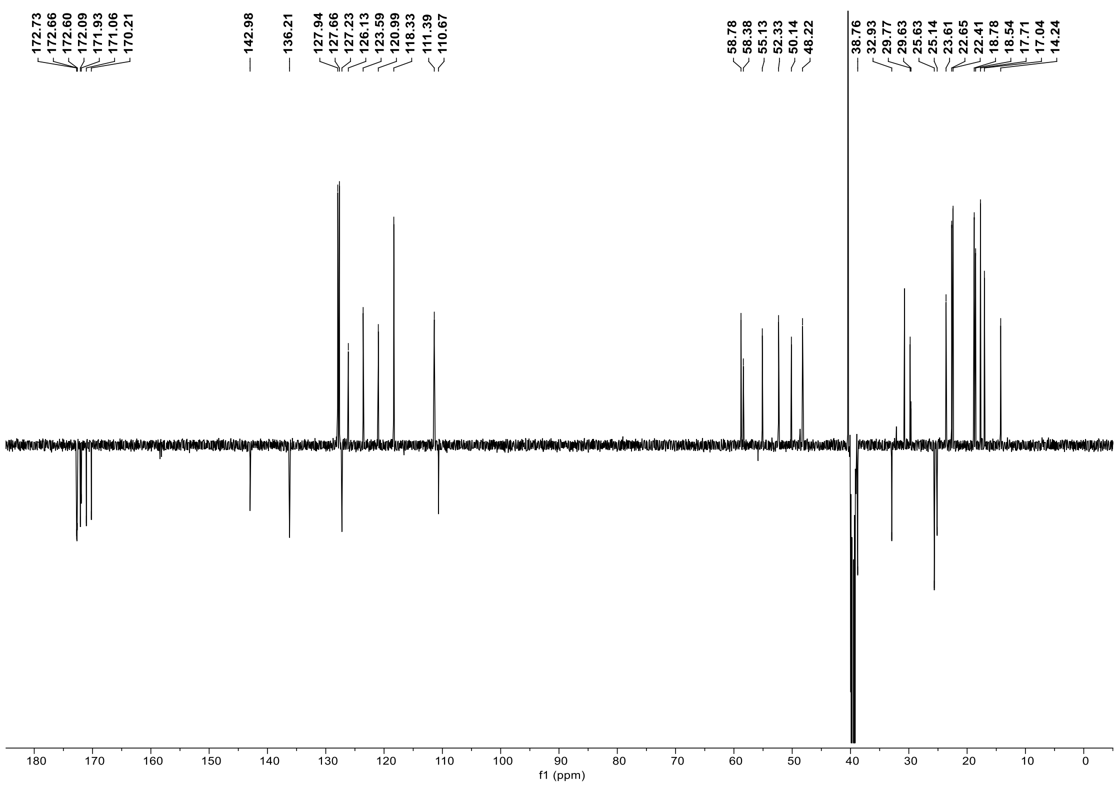

Figure S22. The DEPTQ^{13}C NMR spectrum of 4
